# Enhanced Visualization of Retinal Microvasculature in Optical Coherence Tomography Angiography Imaging via Deep Learning

**DOI:** 10.3390/jcm9051322

**Published:** 2020-05-02

**Authors:** Shin Kadomoto, Akihito Uji, Yuki Muraoka, Tadamichi Akagi, Akitaka Tsujikawa

**Affiliations:** Department of Ophthalmology and Visual Sciences, Kyoto University Graduate School of Medicine, Kyoto 606-8507, Japan; kadomoto@kuhp.kyoto-u.ac.jp (S.K.); muraoka@kuhp.kyoto-u.ac.jp (Y.M.); akagi@kuhp.kyoto-u.ac.jp (T.A.); tujikawa@kuhp.kyoto-u.ac.jp (A.T.)

**Keywords:** OCT angiography, deep learning, averaging, denoising

## Abstract

Background: To investigate the effects of deep learning denoising on quantitative vascular measurements and the quality of optical coherence tomography angiography (OCTA) images. Methods: U-Net-based deep learning denoising with an averaged OCTA data set as teacher data was used in this study. One hundred and thirteen patients with various retinal diseases were examined. An OCT HS-100 (Canon inc., Tokyo, Japan) performed a 3 × 3 mm^2^ superficial capillary plexus layer slab scan centered on the fovea 10 times. A single-shot image was defined as the original image and the 10-frame averaged image and denoised image generated from the original image using deep learning denoising for the analyses were obtained. The main parameters measured were the OCTA image acquisition time, contrast-to-noise ratio (CNR), peak signal-to-noise ratio (PSNR), vessel density (VD), vessel length density (VLD), vessel diameter index (VDI), and fractal dimension (FD) of the original, averaged, and denoised images. Results: One hundred and twelve eyes of 108 patients were studied. Deep learning denoising removed the background noise and smoothed the rough vessel surface. The image acquisition times for the original, averaged, and denoised images were 16.6 ± 2.4, 285 ± 38, and 22.1 ± 2.4 s, respectively (*P* < 0.0001). The CNR and PSNR of the denoised image were significantly higher than those of the original image (*P* < 0.0001). There were significant differences in the VLD, VDI, and FD (*P* < 0.0001) after deep learning denoising. Conclusions: The deep learning denoising method achieved high speed and high quality OCTA imaging. This method may be a viable alternative to the multiple image averaging technique.

## 1. Introduction

Optical coherence tomography angiography (OCTA) is a non-invasive imaging method that images the three-dimensional retinal microvasculature by detecting the motion contrast of blood flow in the retina without intravenous dye injections [1]. OCTA visualizes the retinal microvasculature with higher contrast and better resolution than fluorescein angiography (FA) [2]. The high contrast and resolution in OCTA images make it possible to evaluate the retinal microvasculature quantitatively, including vessel density and nonperfusion areas, more effectively than in FA images [3,4]. 

OCTA artifacts [5] (i.e., motion artifacts) and OCTA scanning protocols [6] (i.e., wide-angle scanning or small A-scan sampling density) easily degrade OCTA image quality, which prevents accurate interpretation and quantitative analysis of OCTA images even in normal populations [7]. Therefore, methods for improving OCTA image quality are important. Uji et al. [8,9,10] and Maloca et al. [11] reported that averaging multiple en face OCTA images improved the OCTA image quality, removing background noise and enhancing the continuity of the vessel, and affected the OCTA data quantitatively and qualitatively. However, substantial image recording time with multiple image acquisitions is a major problem.

Deep learning, which is one of the most common artificial intelligence techniques, reportedly provides a promising solution in image-based medical diagnoses, such as glaucoma and diabetic retinopathy [12,13]. Previous studies have shown that deep learning can enhance the quality of various medical images including ultrasound [14], magnetic resonance imaging [15,16], and optical coherence tomography B-scan images [17]. Since deep learning has the potential to generate a high-quality OCTA image from a single shot image without multiple image acquisition, the application of deep learning to en face OCTA imaging is expected to provide high-quality retinal microvasculature images in a short time. However, to date, OCTA image quality enhancement via deep learning has never been reported.

In this study, we developed a novel deep learning-based algorithm for noise reduction (denoising) in en face OCTA imaging and evaluated the effects of deep learning denoising on the image quality and image acquisition time.

## 2. Materials and Methods

This was a prospective, observational, cross-sectional case series study. The Institutional Review Board of Kyoto University Graduate School of Medicine (Kyoto, Japan) approved this study (000028853), which was conducted according to the tenets of the Declaration of Helsinki. Written informed consent from each subject was obtained before performing any study procedures or examinations.

### 2.1. Participants

Patients with various retinal vascular diseases examined at the Department of Ophthalmology of Kyoto University Hospital between June 2018 and September 2018 were enrolled in the study. All patients underwent a comprehensive ophthalmic examination including measurement of best-corrected visual acuity, slit-lamp biomicroscopy, color fundus photography, and OCTA.

Eyes with keratoconus, high myopia (more severe than -6 diopters or longer than 26.5 mm), or high astigmatism (more severe than ±3 diopters) were excluded. Eyes with OCTA images of poor quality were excluded if significant media opacity was present, if the signal strength was less than 7, or if there were severe motion artifacts (e.g., motion lines).

### 2.2. OCTA Imaging

Each subject was scanned using a spectral-domain OCTA instrument (OCT HS-100; Canon, Inc., Tokyo, Japan). The OCT HS-100 has a scanning rate of 70,000 A-scans/s; a central wavelength of 855 nm; a full-width at half maximum of 100 nm, which enables 3 μm axial resolution in tissue; and a lateral resolution at the retinal surface of 15 μm. The OCT HS-100 scanned the macular area centered on the fovea and measured an area of 3 × 3 mm^2^ (232 × 232 pixels) 10 times with pupil dilation. Then, the 10-frame-averaged en face OCTA images for each subject were created using built-in software in OCT HS-100. The superficial capillary plexus (SCP) was obtained and analyzed using the commercial default automated segmentation boundaries.

Intelligent Denoise (Canon, Inc., Tokyo, Japan), which is a deep learning denoising method developed by Canon, Inc., was utilized to create the denoised en face OCTA image (denoised image). The first en face OCTA image acquisition obtained from 10 scanning sequences was defined as the original image. The original image was processed as the input image, and then the denoised image was automatically output. The system exported three images (original, averaged, and denoised) for further analyses.

The image acquisition time of each image was measured, i.e., the time from when the image acquisition start button was pressed until the viewer displayed the OCTA image.

### 2.3. Network Architecture of Deep Learning Denoising Method and Training Protocol

The U-Net architecture was employed to Intelligent Denoise (Canon, Inc., Tokyo, Japan), which is an encoder–decoder-style neural network that solves semantic segmentation tasks [18]. This network consisted of two parts. Firstly, an encoder took an image tile as input and successively computed feature maps on multiple scales. Secondly, a decoder took the feature representation and classified all pixels/voxels at the original image resolution in parallel. The layers in the decoder synthesized the image, starting at low-resolution feature maps and moving to full-resolution feature maps.

For training, 23,744 datapoints were selected from a set of 742 patients (disease: 595, healthy: 147), which included subjects who received OCTA imaging (OCT HS-100, Canon, Inc., Tokyo, Japan) at Kyoto University Hospital. We labeled the single-shot en face OCTA images as noise patches and the images based on averaged en face OCTA images as denoised patches. Training was performed using a computer with 64 GB of RAM, 4TB HDD, and an NVIDIA 1080Ti 11GB Graphics Processing Unit. The Intelligent Denoise software converted the noisy input en face OCTA images into denoised images.

### 2.4. Quantitative Image Analyses

For objective image quality comparison, the contrast-to-noise ratio (CNR) was calculated as described previously [9,19], using the following equation:

$$\mathrm{CNR}=\left(f-b\right)/\sqrt{\delta_{f}{}^{2}+\delta_{b}{}^{2}},$$

where f and b are the mean gray values of the foreground and background, respectively; and $\delta_{f}$ and $\delta_{b}$ are the standard deviations from the mean values of f and b, respectively. For this calculation, a circular area within the foveal avascular zone (FAZ) was selected as the background region of interest (ROI) and four circular areas at four corners of OCTA image as the foreground ROIs (Supplementary Figure S1). The diameters of these ROIs were 20 pixels (corresponding to areas of about 314 pixels^2^). To match the ROIs among three images (the original, averaged, and denoised images), an ROI manager (https://imagej.nih.gov/ij/developer/api/ij/plugin/frame/RoiManager.html), which recorded the exact locations of the ROIs, was used. The CNR was calculated by automatic execution of ImageJ version 1.52b (National Institutes of Health, Bethesda, MD; https://imagej.nih.gov/ij/index.html) by using a macro that automates a series of ImageJ commands.

Because Intelligent Denoise generated the denoised image using averaged images as training data, the peak signal-to-noise ratio (PSNR), which represents image structural similarity [20], was calculated in the original and denoised images by setting the averaged images as the reference images (ground truths). The definition of the PSNR is

$$\mathrm{PSNR}(\mathrm{dB})=10\log_{10}\left(\frac{MAX_{I}^{2}}{MSE}\right),$$

where MSE stands for mean square error and $MAX_{I}\;$ stands for the greatest potential pixel intensity in image I, which is 255 in the case of an 8-bit grey scale image. A higher PSNR of the sample image indicates good similarity between the ground truth (averaged image) and sample image (original or denoised image). ImageJ calculated the PSNR automatically via the SNR plug-in (http://bigwww.epfl.ch/sage/soft/snr/).

The vessel density (VD), vessel length density (VLD), vessel diameter index (VDI), and fractal dimension (FD) were measured for quantitative analysis of the microvascular density and morphology comparison among the original, averaged, and denoised images in SCP by automatic execution of ImageJ by using a macro. The built-in software in the OCT HS-100 automatically created binarized and skeletonized images and exported them with dimensions of 500 × 500 pixels.

The VD was assessed on the binarized image, defined as the ratio of the area occupied by vessels (white pixels) divided by the total area. The VLD, which represents the length of blood vessels per unit area, was evaluated as described previously [3,9]. The VDI, which represents the average vessel caliber, was calculated by dividing the total vessel area in the binarized image by the total vessel length in the skeletonized image. The FD, which represents the vascular complexity [21,22], was determined on the skeletonized image by using the Box Counting plug-in (https://imagej.nih.gov/ij/plugins/fraclac/FLHelp/BoxCounting.htm). The FD can range from 0 to 2, and images with more complex vessel branching patterns have higher FDs [21].

### 2.5. Expert Comparison of Image Quality

Two experienced ophthalmologists (A.U. and Y.M.) masked to the image information performed independent expert comparisons of original and averaged image pairs or original and denoised image pairs. The ophthalmologists graded 112 pairs of en face OCTA images in total. We arranged the images in two panels (left and right) to facilitate comparison with random assignment of the original versus averaged or original versus denoised images to the left and right panels. The graders assigned scores for comparative image quality between image pairs based on the following three parameters, in line with previous research [9]: (1) vessel quality (contrast and continuity), (2) nonvascular area quality (background noise level), and (3) overall image quality score (overall clarity) to each pair of images. A comparative image quality score was assigned to each image pair as follows: 2 = the left image is definitely better; 1 = the left image is slightly better; 0 = the two images are equal; −1 = the right image is slightly better; and −2 = the right image is definitely better. If the graders disagreed in a particular case, they made an open decision to produce a single determination.

### 2.6. Evaluation of Artifacts in Denoised Images

Deep learning denoising generated two major artifacts in the denoised images. One was “capillary over-dropped out” and the other was “capillary over-generation (pseudo-vessel)”. We arranged the images in two panels (left and right) to facilitate comparison with assignment of the averaged and denoised images to the left and right panels (we set the averaged images as the reference images and placed them on the left side). The two graders (A.U and Y.M) scored the degrees of these two artifacts in the denoised images as follows: 0 = there are no artifacts; −1 = the denoised image has slight artifacts; and −2 = the denoised image definitely has artifacts. If the graders disagreed, they made an open decision to produce a single determination.

### 2.7. Statistical Analyses

Statistical analyses were performed using JMP^®^ 14 (SAS Institute Inc., Cary, NC, USA), presenting all values as the mean ± standard deviation. We compared the differences in the VD, VLD, VDI, FD, and CNR values and image acquisition times for the original, averaged, and denoised images using one-way analysis of variance with the Tukey HSD test for multiple comparison. We assessed the PSNR values obtained from the original and denoised images with paired *t*-tests and analyzed the scores that the two graders provided by performing paired *t*-tests as well. We evaluated the interobserver reproducibility between the two graders by using the kappa statistic κ. We considered *P* values less than 0.05 to be statistically significant.

## 3. Results

Four eyes from five patients that did not meet the inclusion criteria in image quality (signal strength greater than 7) using the 3 × 3 mm^2^ OCTA scan protocol were excluded, leaving 112 eyes from 108 patients for further analyses. The patients had a mean age of 64.2 ± 13.1 years (range: 33–84 years). Among the patients, 60 were male and 48 were female.

The image acquisition times for the original, averaged, and denoised images were 16.6 ± 2.4, 285 ± 38, and 22.1 ± 2.4 s, respectively. The denoised image had a significantly shorter acquisition time than the averaged image (*P* < 0.0001). The ratio between the image acquisition times of the denoised and averaged images was 0.08 ± 0.01, and that between the acquisition times of the denoised and original images was 1.35 ± 0.06.

**Figure 1:**
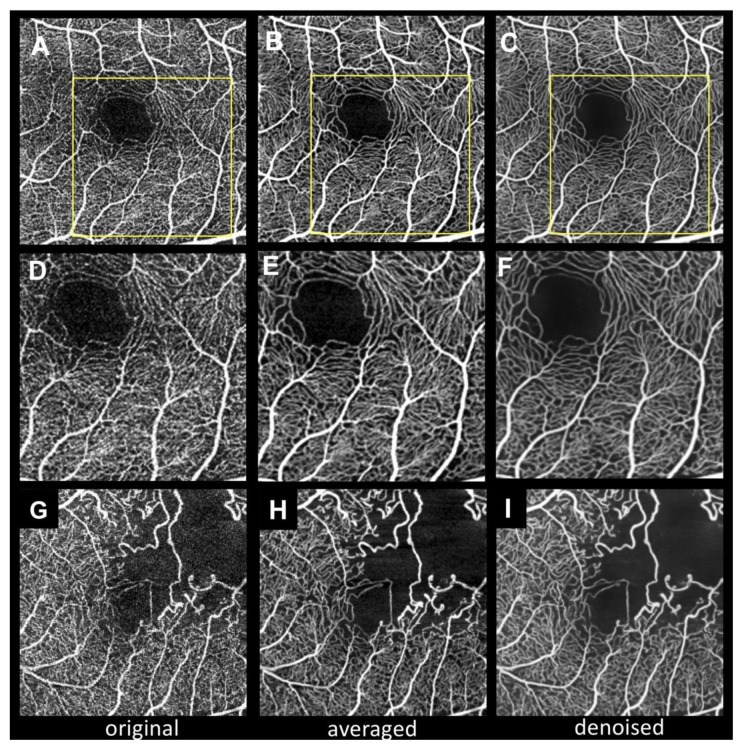
Impact of deep learning denoising on the quality of optical coherence tomography angiography (OCTA) images. (**A**–**C**), OCTA images of normal superficial capillary plexus obtained using a 3 × 3 mm^2^ scan pattern. (**D**–**F**), Magnified images of open squares in A–C. (**G**–**I**), OCTA images of branch retinal vein occlusion. A, D, G, Original OCTA images. B, E, H, Averaged OCTA images. C, F, I, Denoised OCTA images. Averaging and deep learning denoising provide background noise reduction and annealing of disconnected vessels, resulting in noiseless, smooth, and high-contrast images. The contrast-to-noise ratios (CNRs) of A, B, C, G, H, and I are 1.03, 1.42, 1.74, 0.77, 1.16, and 1.27, respectively.

Figure 1 shows representative cases of 3 × 3 mm^2^ SCP OCTA with three different images (original, averaged, and denoised). The averaged image shows more continuous vessels and less background noise than the original image. Notably, the denoised image also shows a high-contrast capillary structure and capillary-free zone around the arteriole, as previous reports have described [23,24]. In contrast, the original image shows fragmented FAZ and noisy capillaries. In the branch retinal vein occlusion case (Figure 1G–I), the averaged and denoised images show well-denoised dots within nonperfusion areas.

As Table 1 demonstrates, the CNR of the denoised image is significantly higher than those of the other two images (*P* < 0.0001). There is no significant difference between the original and averaged images (*P* = 0.0648), although the PSNR of the denoised image is significantly higher than that of the original image (*P* < 0.0001).

Table 2 summarizes the average scores for the subjective image quality assessment. The denoised image scores are significantly higher than those of the averaged images (*P* < 0.0001).

**Figure 2:**
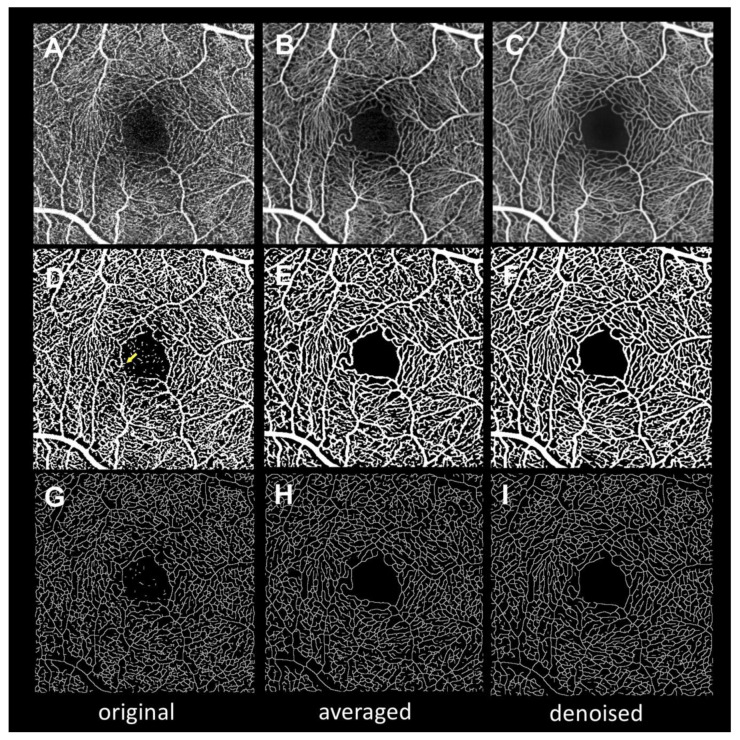
Binarization and skeletonization of en face optical coherence tomography angiography (OCTA) of original, averaged, and denoised images. (**A**–**C**), OCTA images. (**D**–**F**), Binarized images. (**G**–**I**), Skeletonized images. A, D, G, Original images. B, E, H, Averaged images. C, F, I, Denoised images. The binarized image in D shows multiple noise in the foveal avascular zone (FAZ) and burr-like shape at the edges of the vessels (indicated by an arrow), suggesting that background noise could have been falsely included as blood flow. The binarized images of the averaged and denoised images in E and F show less noise and smoother vessels than the original image in D. The skeletonized image in G shows disconnected lines and dots in nonvascular areas. Compared with the original image in G, the skeletonized images of the averaged and denoised images in H and I show annealing lines that reduce the disconnected lines and dots.

Figure 2 presents the images after binarization and skeletonization for use in quantitative measurements. Both the averaged and denoised binarized images show less background noise in FAZ and more continuous vessels than the original binarized images. These findings are also observable in the skeletonized images.

**Figure 3:**
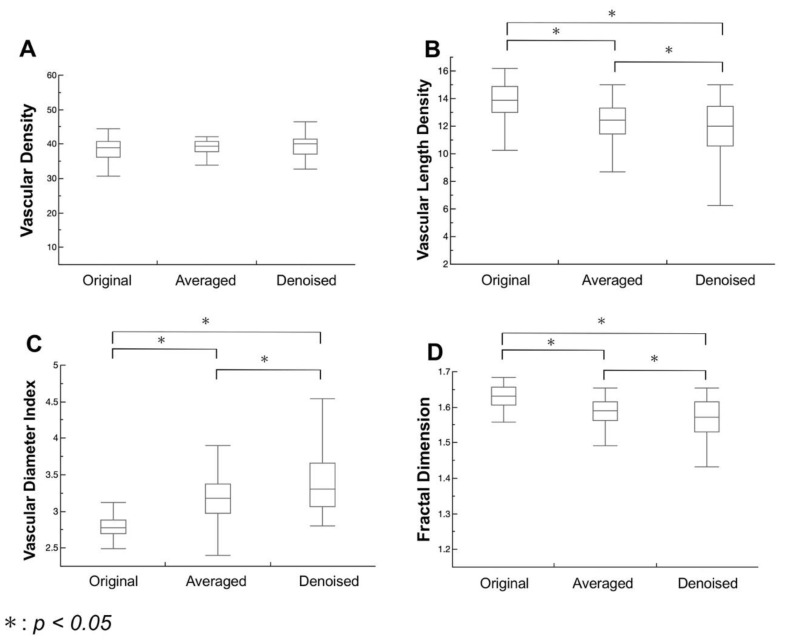
Quantitative analysis of microvascular density and morphology in optical coherence tomography angiography (OCTA) images. Box-whisker plots showing the values of (**A**) vascular density (VD), (**B**) vascular length density (VLD), (**C**) vascular diameter index (VDI), (**D**) and fractal dimension (FD). The horizontal lines represent the maximum (top line), third quartile (top of the box), median (line in the middle of the box), first quartile (bottom of the box), and minimum (bottom line). There are statistically significant differences in VLD, VDI, and FD among the original, averaged, and denoised images. Meanwhile, we found no significant difference in VD among the three images. We utilized *P <* 0.05 in the Tukey–Kramer test.

Figure 3 summarizes the results of quantitative microvascular density and morphology analysis from the binarized and skeletonized images. The three images do not differ significantly in VD (*P* = 0.9199, *P* = 0.4247, and *P* = 0.2307, respectively). The VLD in the denoised image is significantly lower than those in the original and averaged images (*P* < 0.0001, *P* < 0.0001, and *P* = 0.0049, respectively), while the VDI in the denoised image is significantly higher than those in the original and averaged images (*P* < 0.0001). The FD in the denoised image is significantly lower than those in the original and averaged images (*P* < 0.0001).

**Figure 4:**
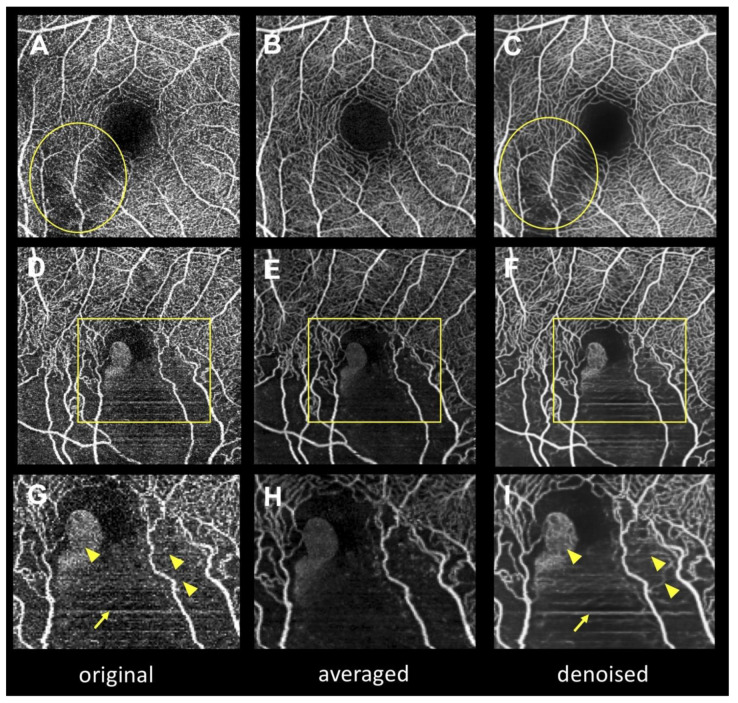
Deep learning-derived artifacts in denoised images. (**A**,**D**,**G**), Original images. (**B**,**E**,**H**), Averaged images. (**C**,**F**,**I**), Denoised images. G–I, Magnified images of open squares in D–F. In the original image in A, signal attenuation is evident in the parafoveal area (open circle) due to media opacity. Capillary over-dropped out artifacts appear in the denoised image in C, which were obtained by applying deep learning denoising. On the other hand, the averaged image in B does not exhibit excessive signal attenuation. Motion artifacts (arrows) and noise accumulation (arrowheads) appear in the original image in G. In the denoised image in I, the motion artifacts appear emphasized (arrow) and the dots have turned into pseudo-vessels due to annealing (arrowheads) (called capillary over-generation), unlike in the averaged image in H.

Figure 4 depicts the artifacts in the denoised images. When there was excessive signal attenuation (i.e., due to the opacity of the medium) in the original en face OCTA image (Figure 4A), capillary over-dropped out artifacts (Figure 4B) occurred in the denoised image. Moreover, deep learning turned aggregated dots into capillary-like structures, producing capillary over-generation (pseudo-vessel, Figure 4H).

Table 3 summarizes the average scores for subjective assessment of artifacts in the denoised images. The average scores in the capillary over-dropped out and capillary over-generation cases are −0.11 ± 0.45 and −0.09 ± 0.41, respectively. Capillary over-dropped out and capillary over-generation artifacts were observed in six and five eyes out of 112 eyes, respectively (5.4% and 4.5%, respectively). The total number of eyes with artifacts (containing either capillary over-dropped out or capillary over-generation artifacts) was 10 out of 112 (8.9%).

## 4. Discussion

In this study, deep learning denoising and multiple image averaging were applied to en face OCTA images. The impact of deep learning denoising on OCTA quantitative parameters and en face OCTA image acquisition times were evaluated and the results were compared with those obtained using the averaging technique. Although there was a significant improvement in the quality in both the denoised and averaged images, deep learning denoising yielded a significantly shorter OCTA image acquisition time than the averaging technique. However, unique deep learning-derived artifacts were observed in the denoised images.

Computed tomography (CT) imaging requires a high radiation dose to obtain high-contrast images, which takes substantial image acquisition time and causes high radiation exposure to patients. Applying deep learning in low-dose-radiation CT imaging [25] can reduce the image acquisition time and radiation exposure. Although OCTA imaging is not an invasive imaging modality, the enormous image acquisition time imposes a certain physical burden on patients and could degrade the image quality, as fatigue resulting from long examination times can cause poor eye fixation and dry eyes. Previous studies have demonstrated that multiple image averaging improves the image quality of en face OCTA by reducing the background noise and enhancing the image contrast [8,10]. However, this approach requires multiple image acquisitions and substantial time. In this study, the image acquisition time with deep learning denoising was observed to be significantly shorter than that with the averaging technique (12.8 ± 1.5 times shorter), and the denoised image acquisition time was statistically determined to be significantly different from the original image (22.1 s vs. 16.6 s *P* < 0.0001). However, this acquisition time in denoising is nominally small and insignificant from a practical standpoint (6 s longer for denoising vs 269 s longer for averaging), suggesting that deep learning has the potential to alleviate the burden on patients of acquiring high-quality OCTA images in patients.

The CNR for image quality assessment was used because distinguishing high bright areas (especially capillaries) from low brightness areas (especially FAZ) is important in OCTA imaging, in which quantitative assessments are based on image binarization [9]. After deep learning denoising, the CNR significantly increased, suggesting the highest image contrast among the original, averaged, and denoised images. In addition, the PSNR, which is one of the most straightforward objective measurements used to compare the similarity between two images, showed higher values in the denoised images than in the original images, suggesting that deep learning denoising can produce high-quality images closer to averaged images. The subjective assessments support these results (overall impression: 1.83 vs. 1.22). In this study, the quantitative parameters describing the microvascular density and morphology (VLD, VDI, and FD) significantly changed after denoising, as previously reported when using the multiple averaging technique [8], suggesting that deep learning denoising could remove the background noise and smooth the rough vessel surface. According to the above results, deep learning denoising can significantly improve the quality of the original OCTA images by reducing background noise and annealing fragmented vessels.

Interestingly, although we developed the deep learning denoising method by using averaged en face OCTA images for training, the CNR and subjective assessments of the denoised images were superior to those of the averaged images. On the contrary, there was no significant difference in CNR between original and averaged images, although a previous study with a small sample size showed a significant difference in CNR between the original and averaged images, where all of the subjects were among the normal population [9]. These findings suggest the possibility that the quality of the averaged images was actually worse than the theoretical estimate (unsuccessful averaging). One possible explanation for this characteristic may be the lower successful image registration rate for multiple image averaging in this study because this study included not only healthy subjects, but also subjects with various retinal diseases, which made it difficult to register each OCTA image due to poor eye fixation (Supplementary Figure S2).

Surprisingly, our study showed no significant difference in VD. Binarization strongly affects the quantification of VD [26]. In this study, the binarization and skeletonized images produced by built-in hardware were used. It could be suggested that the noisy signals and fragmented vessel gaps compensated each other in the original images, as if the process smoothed the pixel counts.

The distributions of each metric in the denoised images were wider than those in the averaged images (Figure 3). Averaging the en face OCTA images facilitated the removal of background noise by equalizing uneven signal distributions. Meanwhile, the denoising method removed background noise by subtracting those pixels that the deep learning denoising method considered as noise even if they were actual flow signals. This result suggested that the denoising method might strongly remove background noise compared with the averaging technique.

We found that two major artifacts occurred in the denoised images: capillary over-dropped out (Figure 4B) and capillary over-generation (pseudo-vessel) artifacts (Figure 4E,H). Ten eyes (8.9%) had artifacts when there was focal signal attenuation or noise accumulation in the original images. Since the purposes of Intelligent Denoise are to reduce background noise and to anneal fragmented vessels, unnatural signal distribution in en face OCTA images (i.e., media opacity shadow or motion artifacts) may be falsely converted into capillary over-dropped out or capillary over-generation artifacts. Although these artifacts always occurred only in small areas of the OCTA images, it is recommended to refer to both original and denoised images for proper interpretation of OCTA images.

This study has several limitations. First, it was not possible to interpret how deep learning enhanced the OCTA image quality because the deep learning algorithm automatically obtained the parameters from learning experience. Second, the sample size of patients was small, which could have made small differences between the groups less detectable. Third, since this study did not evaluate wide-field images exceeding 3 × 3 mm^2^ because the training datasets were based on 3 × 3 mm^2^ en face averaged OCTA images, it is not known whether it would be possible to obtain similar image enhancement in wide-field OCTA images. Fourth, this study only evaluated the superficial vasculature slab because implementing a similar strategy for deeper vessels would be limited by the projection artifact.

In this study, we proposed a novel deep learning denoising method that achieved high-quality OCTA imaging comparable to that provided by multiple image averaging in almost as short of an acquisition time as for a single shot. This deep learning denoising method has the potential to facilitate studies of retinal microvasculature.

## Figures and Tables

**Table 1:** Differences in quantitative metrics among single, averaged, and denoised image in optical coherence tomography angiography images.

Parameter	Original Image	Averaged Image	Denoised Image	*P* Value *	*P* Value †	*P* Value ‡	*P* Value §
Time per Image (s)	16.6 ± 2.4	285 ± 38	22.1 ± 2.4	<0.0001	<0.0001	< 0.0001	NA
Contrast-to-noise ratio	1.092 ± 0.274	1.202 ± 0.331	1.617 ± 0.447	0.0648	<0.0001	< 0.0001	NA
Peak signal-to-noise ratio (dB)	14.3 ± 1.2	NA	17.0 ± 1.3	NA	NA	NA	<0.0001

* Comparison between original and averaged image by the Tukey–Kramer test. † Comparison between original and denoised image by the Tukey–Kramer test. ‡ Comparison between averaged and denoised image by the Tukey–Kramer test. § Comparison between original and denoised image by paired *t* test. NA = not applicable.

**Table 2:** Average scores for subjective image quality assessment of optical coherence tomography angiography images.

	Averaged Image	Denoised Image	*P* Value *	κ in Averaged Images	κ in Denoised Images
Overall impression	1.22 ± 0.61	1.83 ± 0.38	<0.0001	0.965	0.978
Clarity of vessel image	1.17 ± 0.57	1.96 ± 0.19	<0.0001	0.985	0.936
Noise level in avascular area	1.27 ± 0.64	1.92 ± 0.27	<0.0001	0.952	0.782

* Comparison between averaged and denoised image by paired *t* test.

**Table 3:** Average scores for subjective assessment of artifacts in denoised images and numbers of artifacts.

	Scores	Number of Artifacts	κ
Capillary over-dropped out	−0.11 ± 0.45	6/112 (5.4%)	0.955
Capillary over-generation	−0.09 ± 0.41	5/112 (4.5%)	0.985

## Supplementary Materials

The following are available online at https://www.mdpi.com/2077-0383/9/5/1322/s1, Figure S1: Calculation of contrast-to-noise ratio, Figure S2: Representative case of unsuccessful multiple image averaging.

## Abbreviations and Acronyms

optical coherence tomography angiography	OCTA
contrast-to-noise ratio	CNR
peak signal-to-noise ratio	PSNR
vessel density	VD
vessel length density	VLD
vessel diameter index	VDI
fractal dimension	FD
superficial capillary plexus	SCP
foveal avascular zone	FAZ
region of interest	ROI
computed tomography	CT

## References

[1] Jia Y., Tan O., Tokayer J., Potsaid B., Wang Y., Liu J.J., Kraus M.F., Subhash H., Fujimoto J.G., Hornegger J. (2012). Split-spectrum amplitude-decorrelation angiography with optical coherence tomography. Opt. Express.

[2] Spaide R.F., Klancnik J.M., Cooney M.J. (2015). Retinal vascular layers imaged by fluorescein angiography and optical coherence tomography angiography. JAMA Ophthalmol..

[3] Kim A.Y., Chu Z., Shahidzadeh A., Wang R.K., Puliafito C.A., Kashani A.H. (2016). Quantifying Microvascular Density and Morphology in Diabetic Retinopathy Using Spectral-Domain Optical Coherence Tomography Angiography. Investig. Ophthalmol. Vis. Sci..

[4] Kadomoto S., Muraoka Y., Ooto S., Miwa Y., Iida Y., Suzuma K., Murakami T., Ghashut R., Tsujikawa A., Yoshimura N. (2018). Evaluation of Macular Ischemia in Eyes with Branch Retinal Vein Occlusion: An Optical Coherence Tomography Angiography Study. Retina.

[5] Spaide R.F., Fujimoto J.G., Waheed N.K. (2015). Image Artifacts in Optical Coherence Tomography Angiography. Retina.

[6] Rabiolo A., Gelormini F., Marchese A., Cicinelli M.V., Triolo G., Sacconi R., Querques L., Bandello F., Querques G. (2018). Macular Perfusion Parameters in Different Angiocube Sizes: Does The Size Matter in Quantitative Optical Coherence Tomography Angiography?. Investig. Ophthalmol. Vis. Sci..

[7] Lei J., Durbin M.K., Shi Y., Uji A., Balasubramanian S., Baghdasaryan E., Al-Sheikh M., Sadda S.R. (2017). Repeatability and Reproducibility of Superficial Macular Retinal Vessel Density Measurements Using Optical Coherence Tomography Angiography En Face Images. JAMA Ophthalmol..

[8] Uji A., Balasubramanian S., Lei J., Baghdasaryan E., Al-Sheikh M., Sadda S.R. (2017). Impact of Multiple En Face Image Averaging on Quantitative Assessment from Optical Coherence Tomography Angiography Images. Ophthalmology.

[9] Uji A., Balasubramanian S., Lei J., Baghdasaryan E., Al-Sheikh M., Borrelli E., Sadda S.R. (2018). Multiple enface image averaging for enhanced optical coherence tomography angiography imaging. Acta Ophthalmol..

[10] Uji A., Balasubramanian S., Lei J., Baghdasaryan E., Al-Sheikh M., Sadda S.R. (2017). Choriocapillaris Imaging Using Multiple En Face Optical Coherence Tomography Angiography Image Averaging. JAMA Ophthalmol..

[11] Maloca P.M., Spaide R.F., Rothenbuehler S., Scholl H.P.N., Heeren T., Ramos de Carvalho J.E., Okada M., Hasler P.W., Egan C., Tufail A. (2019). Enhanced resolution and speckle-free three-dimensional printing of macular optical coherence tomography angiography. Acta Ophthalmol..

[12] Gargeya R., Leng T. (2017). Automated Identification of Diabetic Retinopathy Using Deep Learning. Ophthalmology.

[13] Liu H., Li L., Wormstone I.M., Qiao C., Zhang C., Liu P., Li S., Wang H., Mou D., Pang R. (2019). Development and Validation of a Deep Learning System to Detect Glaucomatous Optic Neuropathy Using Fundus Photographs. JAMA Ophthalmol..

[14] Perdios D., Besson A., Arditi M., Thiran J.-P. A Deep learning approach to ultrasound image recovery. Proceedings of the 2017 IEEE International Ultrasonics Symposium (IUS).

[15] Hyun C.M., Kim H.P., Lee S.M., Lee S., Seo J.K. (2018). Deep learning for undersampled MRI reconstruction. Phys. Med. Biol..

[16] Lundervold A.S., Lundervold A. (2019). An overview of deep learning in medical imaging focusing on MRI. Z. Für Med. Phys..

[17] Halupka K.J., Antony B.J., Lee M.H., Lucy K.A., Rai R.S., Ishikawa H., Wollstein G., Schuman J.S., Garnavi R. (2018). Retinal optical coherence tomography image enhancement via deep learning. Biomed. Opt. Express.

[18] Ronneberger O., Fischer P., Brox T. U-net: Convolutional networks for biomedical image segmentation. Proceedings of the International Conference on Medical Image Computing and Computer-Assisted Intervention.

[19] Sakamoto A., Hangai M., Yoshimura N. (2008). Spectral-domain optical coherence tomography with multiple B-scan averaging for enhanced imaging of retinal diseases. Ophthalmology.

[20] Zhang K., Zuo W., Chen Y., Meng D., Zhang L. (2017). Beyond a Gaussian Denoiser: Residual Learning of Deep CNN for Image Denoising. IEEE Trans Image Process.

[21] Reishofer G., Koschutnig K., Enzinger C., Ebner F., Ahammer H. (2012). Fractal dimension and vessel complexity in patients with cerebral arteriovenous malformations. PLoS ONE.

[22] Fan W., Nittala M.G., Fleming A., Robertson G., Uji A., Wykoff C.C., Brown D.M., van Hemert J., Ip M., Wang K. (2019). Relationship Between Retinal Fractal Dimension and Non-perfusion in Diabetic Retinopathy on Ultra-Wide Field Fluorescein Angiography. Am. J. Ophthalmol..

[23] Muraoka Y., Uji A., Ishikura M., Iida Y., Ooto S., Tsujikawa A. (2018). Segmentation of the Four-Layered Retinal Vasculature Using High-Resolution Optical Coherence Tomography Angiography Reveals the Microcirculation Unit. Investig. Ophthalmol. Vis. Sci..

[24] Balaratnasingam C., An D., Sakurada Y., Lee C.S., Lee A.Y., McAllister I.L., Freund K.B., Sarunic M., Yu D.Y. (2018). Comparisons Between Histology and Optical Coherence Tomography Angiography of the Periarterial Capillary-Free Zone. Am. J. Ophthalmol..

[25] Akagi M., Nakamura Y., Higaki T., Narita K., Honda Y., Zhou J., Yu Z., Akino N., Awai K. (2019). Deep learning reconstruction improves image quality of abdominal ultra-high-resolution CT. Eur. Radiol..

[26] Mehta N., Liu K., Alibhai A.Y., Gendelman I., Braun P.X., Ishibazawa A., Sorour O., Duker J.S., Waheed N.K. (2019). Impact of Binarization Thresholding and Brightness/Contrast Adjustment Methodology on Optical Coherence Tomography Angiography Image Quantification. Am. J. Ophthalmol..

