# Supplementary material for: Enhanced Visualization of Retinal Microvasculature in Optical Coherence Tomography Angiography Imaging via Deep Learning
Source: J Clin Med. 2020 May 2;9(5):1322. doi: 10.3390/jcm9051322 (PMC7290309; doi:10.3390/jcm9051322)
Supplement: Supplementary file 1 [file jcm-09-01322-s001.pdf]

Journal of Clinical Medicine

Supplemental Figure S1. Calculation of contrast-to-noise ratio.

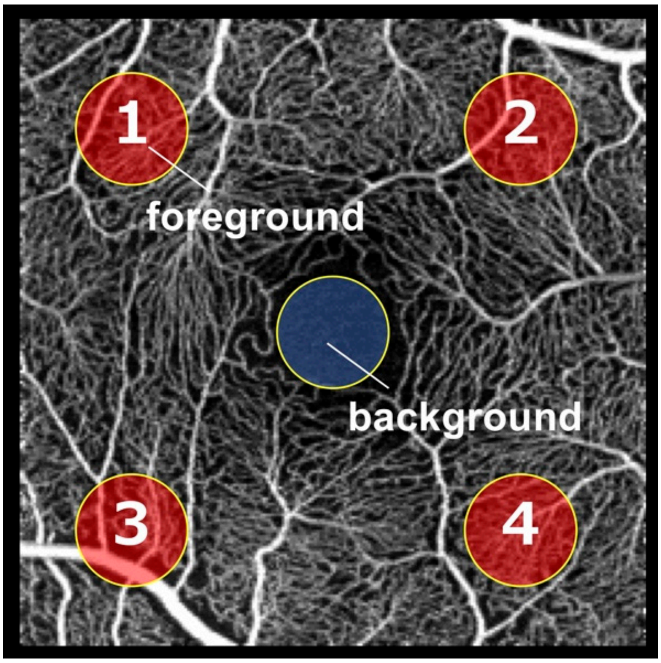

We selected a circular area (blue area) within the foveal avascular zone as the background region of interest (ROI) and four circular areas in the four corners of the OCTA image as the foreground ROIs. The diameters of the circular foreground and background ROIs were 20 pixels (corresponding to areas of about 314 pixels^2).

Supplemental Figure S2. Representative case of unsuccessful multiple image averaging.

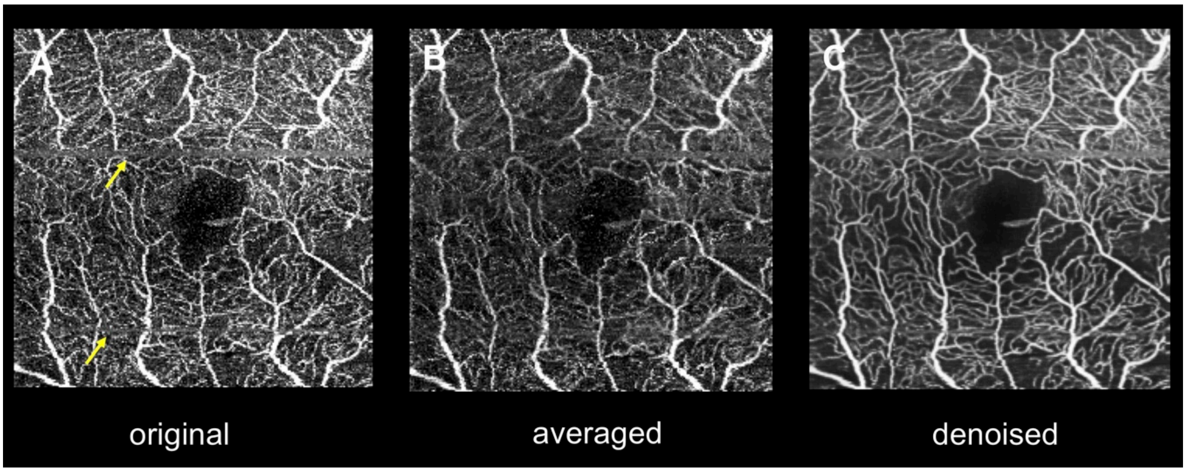

A–C, optical coherence tomography angiography images. A, Original image. B, Averaged image. C, Denoised image. Several motion artifacts (arrows) appear in the original image, indicating that this patient had poor eye fixation. The contrast-to-noise ratios of A, B, and C are 1.04, 0.92, and 1.49, respectively. In this case, the averaged image shows poorer contrast than the original image, whereas the denoised image shows higher contrast than the other two images.
